# Application of data mining methods to improve screening for the risk of early gastric cancer

**DOI:** 10.1186/s12911-018-0689-4

**Published:** 2018-12-07

**Authors:** Mi-Mi Liu, Li Wen, Yong-Jia Liu, Qiao Cai, Li-Ting Li, Yong-Ming Cai

**Affiliations:** 1 School of Public Health, Guangdong Pharmaceutical University, Guangzhou, Guangdong China; 2 School of Clinical Medicine, Guangdong Pharmaceutical University, Guangzhou, Guangdong China; 3 College of Medical Information Engineering, Guangdong Pharmaceutical University, Guangzhou, Guangdong China; 4 Guangdong Chinese Medicine Big Data Engineering Research Center, Guangzhou, Guangdong China

**Keywords:** C5.0 decision tree, Tree augmented naive bayesian network, Multilayer perceptron, Logistic regression, SMOTE, Early gastric cancer, Stomach neoplasms

## Abstract

**Background:**

Although gastric cancer is a malignancy with high morbidity and mortality in China, the survival rate of patients with early gastric cancer (EGC) is high after surgical resection. To strengthen diagnosing and screening is the key to improve the survival and life quality of patients with EGC. This study applied data mining methods to improve screening for the risk of EGC on the basis of noninvasive factors, and displayed important influence factors for the risk of EGC.

**Methods:**

The dataset was derived from a project of the First Hospital Affiliated Guangdong Pharmaceutical University. A series of questionnaire surveys, serological examinations and endoscopy plus pathology biopsy were conducted in 618 patients with gastric diseases. Their risk of EGC was categorized into low and high risk of EGC by the results of endoscopy plus pathology biopsy. The synthetic minority oversampling technique (SMOTE) was used to solve imbalance categories of the risk of EGC. Four classification models of the risk of EGC was established, including logistic regression (LR) and three data mining algorithms.

**Results:**

The three data mining models had higher accuracy than the LR model. Gain curves of the three data mining models were convexes more closer to ideal curves by contrast with that of the LR model. AUC of the three data mining models were larger than that of the LR model as well. The three data mining models predicted the risk of EGC more effectively in comparison with the LR model. Moreover, this study found 16 important influence factors for the risk of EGC, such as occupations, helicobacter pylori infection, drinking hot water and so on.

**Conclusions:**

The three data mining models have optimal predictive behaviors over the LR model, therefore can effectively evaluate the risk of EGC and assist clinicians in improving the diagnosis and screening of EGC. Sixteen important influence factors for the risk of EGC were illustrated, which may helpfully assess gastric carcinogenesis, and remind to early prevention and early detection of gastric cancer. This study may also be conducive to clinical researchers in selecting and conducting the optimal predictive models.

## Background

Gastric cancer is a common malignancy with high incidence and mortality in China. According to the latest statistical report, the incidence of it in 2013 was the second highest after liver cancer (31.38 patients with gastric cancer per 100,000 people), and the number of deaths from gastric cancer was third [1]. In China, the incidence and mortality of gastric cancer is much higher than that of developed and other developing countries, and gastric cancer will be the primary reason of malignant tumors deaths by 2020 [2–4]. Surgical resection is considered to be the radical treatment of early gastric cancer (EGC), and the postoperative 5-year survival rate of EGC should be 90%. Therefore, it is important for patients to strengthen diagnosing and screening of EGC. However, the EGC patients usually have no specific symptoms, and a few symptoms of EGC are similar to that of gastritis or dyspepsia, hence EGC is easy to be ignored by the patients. When the patients have obvious symptoms, most of them have developed into advanced gastric cancer; although the patients with advanced gastric cancer receive treatment, the 5-year survival rate of them decreases to only 30–40% [5].

Most scholars believe that endoscopy plus pathology biopsy is the gold standard in the screening of EGC. However, owing to unpopularity and low compliance of endoscopy plus pathology biopsy, the detection rate of EGC is low in China [6, 7]. The purpose of this study was to construct prediction models to screen the risk of EGC based on noninvasive factors, such as demographic characteristics, eating habits, main symptoms during the nearly 3 months, family or previous diseases histories and serological examinations of the patients with gastric diseases, and analyze the great influences on the risk of EGC simultaneously, so that assist clinical decisions-making to elevate screening for the risk of EGC further.

## Methods

### Subjects

The subjects of this study came from a project —“An Innovative Platform of Screening Early Gastric Cancers based on Cloud Computing” in the First Hospital Affiliated Guangdong Pharmaceutical University. From January 2016 to May 2017, a total of 620 patients with gastric diseases agreed to participate in the project, they were hospitalized at digestive system department of 26 hospitals involved in the project. The participants filled out a questionnaire, including nine demographic characteristics, 11 eating habits, 14 main symptoms during the nearly 3 months and nine family or previous diseases histories. Their results of 5 serological examinations and endoscopy plus pathology biopsy were recorded, the latter is the gold standard in the screening of EGC. The data type of the above 48 items from questionnaires and serological examinations were different, such as discrete numerical, continuous numerical and categorical, besides these items had complicated relationships each other. Two participants who were diagnosed with gastric cancer were excluded, so 618 participants were eventually included in the original dataset. The 618 participants were classified into low risk of EGC (487 cases) and high risk of EGC (131 cases) in accordance with their results of endoscopy plus pathology biopsy. A correlation analysis was conducted, consequently 14 items having weak correlation with the risk of EGC were eliminated. Finally, when the prediction models of the risk of EGC were established, the remaining 34 items as the influence factors for the risk of EGC are independent variables and the risk of EGC was dependent variable.

### Processing the datasets

By a stratified random sampling based on the risk of EGC, the original dataset were partitioned into 70% training set and 30% testing set. Training set was used to generate a model and testing set to evaluate the model finally, then we were likely to get a good indication of how well the model would generalize to other datasets that were similar to the current dataset [8].

The proportion of low and high risk of EGC on the training set was imbalanced (patients at low and high risk of EGC is successively 344 cases and 98 cases). The imbalance of classification would decrease the predictive performance of classifiers, so the current study used the synthetic minority oversampling technique (SMOTE) to balance the training set. SMOTE is different from simple oversampling with replacement and undersampling. Since simple oversampling with replacement excessively uses the original dataset, models may have low generalization. However, undersampling possibly results in inaccurate models for not taking full advantage of the original dataset [9, 10]. SMOTE produces synthetic data between a minority sample and its nearest neighbors based on a distance calculated by standard Euclidean distance between minority samples, which avoids the above problems caused by simple oversampling with replacement and undersampling [11]. Some previous researches have indicated that SMOTE effectively accelerated the accuracy of classifiers, such as support vector machine, C4.5 decision tree, random forest, Bayesian network and neural network [12–15]. After handling the imbalanced classification with SMOTE, the samples of the training set increased to 516 cases, with 344 cases at low risk of EGC and 172 cases at high risk of EGC. The oversampled training set was used for establishing the prediction models.

### Building risk prediction models

C5.0 decision tree (C5.0 DT) algorithm generates well-understood classification rules, even though the independent variables possess complex relationships each other. The C5.0 algorithm improves its accuracy significantly by the boosting method. Boosting works by building multiple models in a sequence. The first model is built in the usual way. Then, a second model is built in such a way that it focuses on the cases that were misclassified by the first model. Then a third model is built to focus on the errors of the second model, and so on. Finally, cases are classified by applying the whole set of models to them, using a weighted voting procedure to combine the separate predictions into one overall prediction. C5.0 DT not only is robust in the processing of high-dimensional data, but also has high execution efficiency, so it is applicable to the classification of big data [16, 17]. To prevent overtraining C5.0 DT by reason of unavoidable noisy, this study adopted a series of measures as follows: setting pruning severity to 85%, making 10 samples as minimum samples per child branch of the tree and choosing global pruning method to optimize the tree globally.

The tree augmented naive Bayesian network (TAN), a simple Bayesian network that is an improvement over the standard Naive Bayes model, allows each independent variable to depend on another independent variable apart from the dependent variable, thereby increasing the classification accuracy [18]. In current study, the parameters learning method of TAN was Bayes adjustment that was suitable for small datasets and applied smoothing to reduce the effect of any zero-counts and any unreliable estimate effects, these parameters were used to estimate the conditional probability tables among variables. Finally, likelihood ratio was applied to independence tests between independent variables and dependent variable.

Neural networks, simplified models of the way by which the human brain processes information, work by simulating enormous interconnected processing units that resemble abstract neurons. This study performed a multilayer perceptron (MLP), despite possibly took more time to train and score. MLP dealed with more complex relationships and had increased predictive power compared to the radial basis function algorithm [19]. This study created a standard MLP model, which was easy to interpret and fast to score, rather than an ensemble model that used boosting to obtain more accurate predictions or used bagging to obtain more reliable predictions.

Logistic regression model (LR) estimates probability of each sample belonging to a certain category, and the target category with the highest probability is assigned as the prediction result for that sample. Because the dependent variable, the risk of EGC, had two categories, a binomial LR was established in this study. The forwards stepwise method was devoted to just including important influence factors in the risk prediction model of EGC. This study set low risk of EGC as the base category of the LR model, and the other modeling options were the defaults.

### Evaluation and comparison of the models

This study evaluated and compared the predictive performance of the four models in terms of confusion matrix, classification accuracy, AUC and gains, all these were based on testing set. Accuracy is the percentage of the samples correctly classified accounting for the total samples. AUC represents the area under the receiver operator characteristic curve. Gains are defined as the proportion of total hits that occurred in each quantile, that is to say, they were computed as (number of hits in quantile/total number of hits) × 100%.

All processes of this study, including analyzing the correlation between the 48 items and the risk of EGC, splitting the original dataset into two parts, oversampling the training set with SMOTE, and creating, analyzing and evaluating the four prediction models, were performed in the software SPSS Modeler, version 18.1.0.

## Results

### Characteristics of the subjects

The demographic characteristics, eating habits, main symptoms during the nearly 3 months, family or previous diseases histories and serological examinations of the 618 participants are displayed in Tables 1, 2, 3, 4 and 5. As outlined in Table 1, the proportion of workers at high risk of EGC was higher when compared with that of workers at low risk of EGC (47.33% versus 37.56%); patients with gastric diseases who spoke cantonese were the primary population at high risk of EGC (45.80%). Among the patients at high risk of EGC, 71.76% of them seldom drank tea and 51.91% of them preferred drinking hot water, both were significantly more than the patients at low risk of EGC (Table 2). The number of patients having the main symptoms during the nearly 3 months of acid reflux, belching and ostprandial distress increased with the risk of EGC, as exhibited in Table 3. Table 4 shows that the patients at high risk of EGC had more family histories of hyperlipidemia or had more positive helicobacter pylori (HP) infection than those at low risk of EGC. In Table 5, 45 point zero 3 % of the patients at high risk of EGC were tested positive or weakly positive for HP antibody, whereas that of the patients at low risk of EGC was 35.31%.

**Table 1:** The demographic characteristics of the participants

	Low risk of EGC	High risk of EGC
	(*n* = 487)	(*n* = 131)
Sex		
Male	237 (48.67)	65 (49.62)
Female	250 (51.33)	66 (50.38)
Age (year)^a^	51.36 (11.49)	53.37 (10.75)
Weight (kg)^a^	59.43 (9.54)	58.84 (9.77)
Height (cm)^a,b^	161.99 (7.57)	161.68 (7.31)
BMI^a^	22.61 (3.00)	22.43 (2.81)
Education levels		
Illiterate	10 (2.05)	1 (0.76)
Primary school	97 (11.92)	34 (25.95)
Junior school	156 (32.03)	47 (35.88)
Senior school	116 (23.82)	22 (16.79)
College	108 (22.18)	27 (20.62)
Occupations		
Cadre	162 (33.26)	44 (33.59)
Worker	183 (37.58)	62 (47.33)
Peasant	142 (29.16)	25 (19.08)
Languages		
Mandarin	71 (14.58)	20 (15.27)
Cantonese	154 (31.62)	60 (45.80)
Hakka	161 (33.06)	34 (25.95)
Teochew	101 (20.74)	17 (12.98)
Residences		
City	217 (44.56)	57 (43.51)
Townlet	142 (29.16)	30 (22.90)
Village	128 (26.28)	44 (33.59)

^a^Data are presented as a mean (SD), others are presented as a number (percentage)

^b^Items were eliminated because of weak correlation with the risk of EGC

**Table 2:** The eating habits of the participants

	Low risk of EGC	High risk of EGC
	(*n* = 487)	(*n* = 131)
High salt intake		
Yes	137 (28.13)	39 (29.77)
No	350 (71.87)	92 (70.23)
Pickled foods		
Often	57 (11.70)	16 (12.21)
Seldom	430 (88.30)	115 (87.79)
Fried/smoke foods^a^		
Often	43 (8.83)	6 (4.58)
Seldom	444 (91.17)	125 (95.42)
Fruit		
Often	240 (49.28)	75 (57.25)
Seldom	247 (50.72)	56 (42.75)
Vegetable^a^		
Often	456 (93.63)	128 (97.71)
Seldom	31 (6.37)	3 (2.29)
Tea		
Often	168 (34.50)	37 (28.24)
Seldom	319 (65.50)	94 (71.76)
Smoking		
Yes	149 (30.60)	43 (32.82)
No	338 (69.40)	88 (67.18)
Drinking		
Yes	79 (16.22)	21 (16.03)
No	408 (83.78)	110 (83.97)
Drinking-water source		
Water supply	422 (86.65)	124 (94.66)
Wells water	50 (10.27)	7 (5.34)
Rivers water	15 (3.08)	0 (0.00)
Drinking hot water		
Yes	204 (41.89)	68 (51.91)
No	283 (58.11)	63 (48.09)
Speed of eating		
Fast	306 (62.83)	70 (53.44)
Slow	181 (37.17)	61 (46.56)

All data are presented as a number (percentage)

^a^Items were eliminated because of weak correlation with the risk of EGC

**Table 3:** The main symptoms during the nearly 3 months of the participants

	Low risk of EGC	High risk of EGC
	(*n* = 487)	(*n* = 131)
Abdominal pain		
Yes	228 (46.82)	64 (48.85)
No	259 (53.18)	67 (51.15)
Abdominal distension		
Yes	220 (45.17)	66 (50.38)
No	267 (54.83)	65 (49.62)
Acid reflux		
Yes	143 (29.36)	48 (36.64)
No	344 (70.64)	83 (63.36)
Belching		
Yes	125 (25.67)	40 (30.53)
No	262 (74.33)	91 (69.47)
Early satiety		
Yes	57 (11.70)	19 (14.50)
No	430 (88.30)	112 (85.50)
Postprandial distress		
Yes	91 (18.69)	31 (23.66)
No	396 (81.31)	100 (76.34)
Heartburn		
Yes	61 (12.53)	22 (16.79)
No	426 (87.47)	109 (83.21)
Melaena^a^		
Yes	36 (7.39)	9 (6.87)
No	451 (92.61)	122 (93.13)
Emaciation^a^		
Yes	37 (7.60)	7 (5.34)
No	450 (92.40)	124 (94.66)
Poor appetite^a^		
Yes	39 (8.01)	9 (6.87)
No	448 (91.99)	122 (93.13)
Dysphagia^a^		
Yes	6 (1.23)	3 (2.29)
No	481 (98.77)	128 (97.71)
Nausea^a^		
Yes	42 (8.62)	14 (10.69)
No	445 (91.38)	117 (89.31)
Poststernal discomfort^a^		
Yes	44 (9.03)	16 (12.21)
No	443 (90.97)	115 (87.79)
No obvious symptom		
Yes	56 (11.50)	16 (12.21)
No	431 (88.50)	115 (87.79)

All data are presented as a number (percentage)

^a^Items were eliminated because of weak correlation with the risk of EGC

**Table 4:** The family or previous diseases histories of the participants

	Low risk of EGCs	High risk of EGCs
	(*n* = 487)	(*n* = 131)
Esophageal cancer^a^		
Yes	14 (2.87)	2 (1.53)
No	473 (97.13)	129 (98.47)
Gastric cancer^a^		
Yes	25 (5.13)	9 (6.87)
No	462 (94.87)	122 (93.13)
Colorectal cancer^a^		
Yes	8 (1.64)	3 (2.29)
No	477 (98.36)	128 (97.71)
Diabetes mellitus^a^		
Yes	30 (6.16)	14 (10.69)
No	457 (93.84)	117 (89.31)
Hypertension		
Yes	78 (16.02)	19 (14.50)
No	409 (83.98)	112 (85.50)
Hyperlipidemia		
Yes	68 (13.96)	27 (20.61)
No	419 (86.04)	104 (79.39)
HP infection		
Negative	23 (4.72)	12 (9.16)
Positive	29 (5.95)	17 (12.98)
Unidentified	435 (89.32)	102 (77.86)
Gastroscopy		
Yes	96 (19.71)	25 (19.08)
No	391 (80.29)	106 (80.92)
Gastric ulcer^a^		
Yes	28 (5.75)	10 (7.63)
No	459 (94.25)	121 (92.37)

All data are presented as a number (percentage)

^a^Items were eliminated because of weak correlation with the risk of EGC

**Table 5:** The serological examinations of the participants

	Low risk of EGC	High risk of EGC
	(*n* = 487)	(*n* = 131)
Pepsinogen I (ug/L)^a^	139.18 (94.03)	140.32 (91.61)
Pepsinogen II (ug/L)^a^	16.68 (27.80)	17.26 (23.95)
Gastrin 17 (pmol/L)^a^	8.04 (13.72)	8.67 (16.18)
Pepsinogen I/II^a^	12.74 (6.19)	12.35 (6.55)
HP antibody		
Negative	315 (64.68)	72 (54.96)
Weakly positive	55 (11.29)	19 (14.50)
Positive	117 (24.02)	40 (30.53)

^a^Data are presented as a mean (SD), others are presented as a number (percentage)

### Modeling results

After trained by the training set, a C5.0 DT model with 10 base decision trees was built, the 10 base decision trees were corresponding 10 sets of intelligible classification rules. Taking one base decision tree as an example, one leaf of it had the corresponding classification rule as follows: IF one participant often ate pickled foods, AND he/she had weakly positive HP antibody in serum, AND his/her drinking-water was wells water, THEN his/her probability at low risk of EGC was 81.82%, and at high risk of EGC was 18.18%. If a participant fit in the above rule, he/she would be classified as low risk of EGC by this decision tree. Similarly, another nine base decision trees alternately classified the same participant as a certain risk of EGC according to their classification rules. Finally, the C5.0 DT model chose ensemble predicted values for this participant by using voting. Voting selected the category that most often had higher probability across the 10 base decision trees.

The TAN model was a probability network that revealed the conditional probability for each independent variable and dependent variable. The conditional probability table of each variable was output, it contained the conditional probability value for each variable value and each combination of values in its parent variables. These conditional probability tables were integratively used to predict the probability of participants at each risk of EGC, ultimately the TAN model selected the category that achieved the highest probability.

The MLP model possessing three parts: input layer, hidden layer and output layer, was too complex to be explain easily. In this paper, despite the interpretability of model, the MLP model exactly predicted the risk of EGC, and its accuracy was 77.84% in Table 6. Clinical scholars believed that when patients at low risk of EGC were diagnosed mistakenly at high risk of EGC would result in medical resources waste; however, misdiagnosing patients at high risk of EGC maybe lead to miss the optimal cure time, and the patients misdiagnosed would pay heavy prices, even die in severe case. Thus the high risk of EGC is usually the critical class, which investigator tend to predict it with higher accuracy. Fifteen patients at high risk of EGC were accurately predicted by the MLP model more than the other three models, as shown in Table 6. The higher accuracy of the MLP model for predicting the high risk of EGC was in conformity with the above clinical practice.

**Table 6:** The confusion matrix, accuracy and AUC of the four models on testing set

	Confusion matrix			Accuracy(%)	AUC
		L	H		
C5.0 DT	L	129	14	77.84	0.66
	H	25	8		
TAN	L	127	16	77.27	0.65
	H	24	9		
MLP	L	122	21	77.84	0.74
	H	18	15		
LR	L	120	23	73.30	0.62
	H	24	9		

Confusion matrix shows the number of cases at each risk of EGC on the testing set. In confusion matrix, the columns denote the actual risk of EGC and the rows denote the predicted; L and H respectively stand for low risk of EGC and high risk of EGC

The LR model consisted of a equation by reference to the base category, the low risk of EGC, the probability of participants at each risk of EGC could be calculated from the equation. The LR model exhibited odds ratios of high risk of EGC compared with the base category, and the predicted probabilities of each sample was obtained from those odds ratios. What did come out, the sample was of membership for a certain risk of EGC that achieved the higher predicted probability. As a result of this study, all of the 34 independent variables were selected to establish the probability equation by the forwards stepwise method.

### Importance of independent variables

In SPSS Modeler software, the four models clarified the relative importance of each independent variables for classifying the dependent variable. In descending order of total importance, the independent variables and their importance were illustrated in Table 7. The importance of independent variables calculated by C5.0 DT and MLP were similar, it was difficult to distinguish the most influential independent variables for the prediction models. But TAN and LR did the opposite, especially LR, the importance of independent variables was obvious gradient, it indicated that even dealing with high dimensional data, the LR model effectively picked out important independent variables. In general, the total importance of all influence factors for the risk of EGC were also displayed (see Table 7), the 16 most important influence factors were occupations, HP infection, HP antibody, weight, drinking-water source, age, pepsinogen I, gastrin 17, education levels, residences, BMI, pepsinogenI/II, languages, tea, drinking hot water and gastroscopy, their total importance were higher than the mean value of all total importance.

**Table 7:** Important independent variables for the risk of EGC

Variables	C5.0 DT	TAN	MLP	LR	Total
Occupations	0.03	0.10	0.04	0.04	0.21
HP infection	0.03	0.05	0.04	0.09	0.21
HP antibody	0.03	0.02	0.04	0.11	0.20
Weight	0.03	0.08	0.07	0.02	0.20
Drinking-water source	0.03	0.04	0.03	0.06	0.16
Age	0.03	0.03	0.06	0.03	0.15
Pepsinogen I	0.03	0.02	0.08	0.02	0.15
Gastrin 17	0.04	0.02	0.07	0.02	0.15
Education levels	0.03	0.02	0.03	0.05	0.13
Residences	0.03	0.04	0.02	0.04	0.13
BMI	0.03	0.03	0.04	0.02	0.12
PepsinogenI/II	0.03	0.02	0.05	0.02	0.12
Languages	0.03	0.02	0.03	0.04	0.12
Tea	0.03	0.06	0.01	0.02	0.12
Drinking hot water	0.03	0.03	0.02	0.04	0.12
Gastroscopy	0.03	0.03	0.03	0.03	0.12
High salt intake	0.03	0.05	0.01	0.02	0.11
Abdominal pain	0.03	0.03	0.02	0.03	0.11
Hypertension	0.03	0.03	0.03	0.02	0.11
Hyperlipidemia	0.03	0.03	0.03	0.02	0.11
Smoking	0.03	0.03	0.02	0.02	0.10
Heartburn	0.03	0.02	0.03	0.02	0.10
Pepsinogen II	0.03	0.02	0.03	0.02	0.10
Fruit	0.03	0.02	0.02	0.02	0.09
Acid reflux	0.03	0.02	0.02	0.02	0.09
Postprandial distress	0.02	0.02	0.03	0.02	0.09
Speed of eating	0.03	0.03	0.02	0.01	0.09
Abdominal distension	0.03	0.01	0.02	0.03	0.09
Drinking	0.03	0.02	0.01	0.02	0.08
Sex	0.03	0.02	0.01	0.01	0.07
Pickled foods	0.03	0.01	0.01	0.02	0.07
Early satiety	0.03	0.01	0.01	0.02	0.07
Belching	0.03	0.01	0.01	0.01	0.06
No obvious symptom	0.01	0.01	0.01	0.02	0.05

The sum of the 34 independent variables’ importance calculated by each model is equal to one. The sum of the 34 independent variables’ total importance is 4

### Performance results

**Fig. 1:**
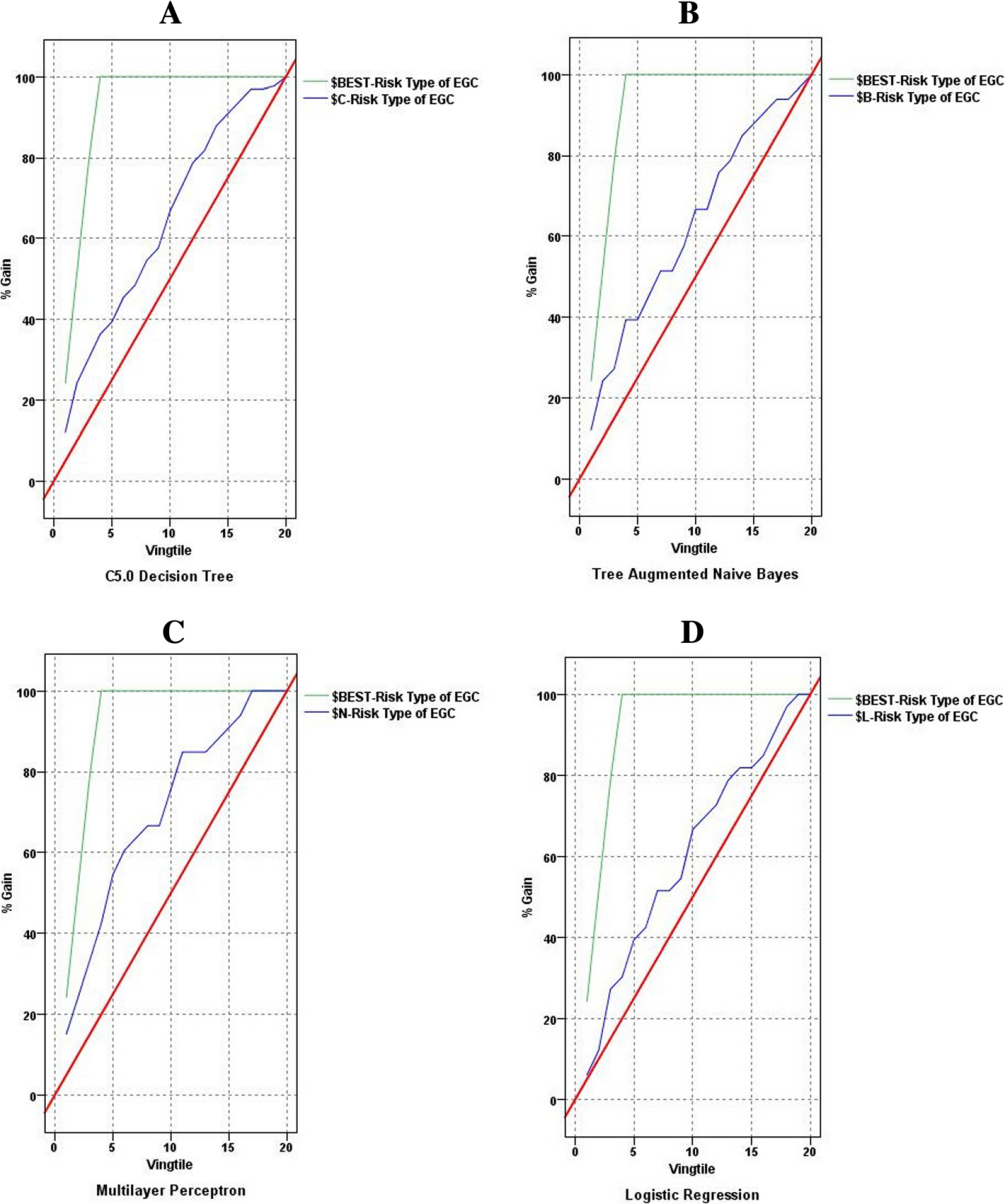
The gains charts of the four models. The top polygonal line is ideal curve, and the irregular curve is gains curve of a model between the ideal curve and the diagonal. For a good model, the gains curve will rise steeply toward 100% and then level off. A model that provides no predictive performance will follow the diagonal from lower left to upper right. As shown in this figure, the gain curves of the three data mining models (image **a**, **b** and **c**) were convexes close to the ideal curves, especially the MLP model. However, the gain curve of the LR model (image **d**) rose slowly away from the ideal curve

Table 6 points out the confusion matrix, accuracy and AUC of the four models on testing set. The gains charts of the four models are illustrated in Fig. 1. The models of C5.0 DT, TAN and MLP had higher accuracy than the LR model. In the gains chats of the three data mining models, the gain curves were convexes more closer to the ideal curves by contrast with the LR model. The gain curve in the gains chart of the LR model rose slowly away from the ideal curve. AUC of the three data mining models were larger than that of the LR model as well. It indicated that the LR model did not classify the risk of EGC effectively when compared to the three data mining models. As known from the confusion matrix, the MLP model considered its clinical translation and was in accordance with the clinical practice of screening EGC, because it was biased towards classifying as high risk of EGC. A model that is biased towards classifying as high risk may be “better” than one that biases towards low risk classifications, given the consequences of missing the cancer diagnosis. Furthermore, the MLP model had the largest AUC, it revealed that the MLP model had the best classification effect among the three data mining models.

## Discussion

### Performance evaluation and comparison

Comparing with the other three models, classification rules produced by the C5.0 DT model are easier to understand and apply in clinical practice. The TAN model shows the distribution of conditional probabilities, which commendably interprets the probabilistic dependency relationships between independent variables and dependent variable. The LR model was effective in previous traditional epidemiological and health statistical studies, and it calculated odds ratios relative to the base category. However, when the LR model was applied to processing the big or high-dimensional data, it was less effective contrasting with data mining models. As this study, consequently the three data mining models had more hopeful classification effects in comparison with the LR model, which effectively improved screening for the risk of EGC, especially the MLP model which with the highest accuracy, the largest AUC and consideration of the classfier’s clinical translation.

Although the traditional statistical models easily explain the relationship between dependent variables and independent variable, they fail to cope with enormous variables, various types of variables and complex relationships among variables [20–22]. If the purpose of one research is to boost the performance of prediction models, and the interpretability of models is secondary, then researchers prefer to develop data mining models to obtain gratifying predictions [23]. Therefore, the above discussion may fully clarify that the three data mining models are potentially optimal models of improving screening for the risk of EGC, the MLP model in especial.

### Important independent variables

This study sought out 16 important influence factors for the risk of EGC, they may be of crucially considerable value in screening the risk of EGC. When focusing on the 16 factors, clinicians can rapidly evaluate which risk of EGC the patients with gastric disease at. The 16 factors involve four serological examinations: HP antibody, pepsinogen I, gastrin 17 and pepsinogenI/II, it suggests that serological examinations are of the important methods for screening the risk of EGC. Yamaguchi Y also found that a ABC method, which combined assay of HP and serum pepsinogen, was useful for screening gastric cancer in high-risk and low-risk populations [24]. Many epidemiological researchers has reported that HP infection is a risk factor for gastric cancer. HP participate in invasion, metastasis and clinical stage of gastric cancer, and it promote the pathogenesis of gastric cancer, so it is clinically a potential marker for evaluating the progress and prognosis of gastric cancer [25, 26].

This study indicates that drinking-water sources is a important factor for the risk of EGC. Wells and rivers water may be contaminated due to lacking of effective regulations, the pollution sources include industrial waste, agricultural fertilizers and pesticides, and microorganisms [27–29]. The wells and rivers water polluted as drinking water should cause gastrointestinal malignant tumors, which may be closely related to the following factors: bacteria, cyanotoxins, sulfates, nitrates, minerals, microelements, chlorides, heavy metals and so on [30].

Many eating habits importantly affect the risk of EGC as well. On the one hand, previous studies have found that people who frequently drink tea and eat fruits had low rate of tumors [31, 32]. On the other hand, there are dangerous eating habits, such as often drinking hot water. Constantly drinking hot water induces mucosal injuries in the digestive tract, which accelerate the carcinogenic processes of carcinogens [33]. It suggest that people drink less hot water to prevent gastric cancers. Though previous researchers deemed that smoking and drinking likely cause a variety of cancers, this study did not take them as important factors of the risk of EGC, potentially on account of no quantitatively analyzing smoking and drinking [34, 35].

The four demographic characteristics: occupations, residences, education levels and languages, imply the social status and health care consciousness of the participants, which may further determine their eating habits and so on, so this four demographic characteristics have comprehensive effects on the patients in respect of their risk of EGC. Some studies had shown that family history of gastric cancer was risk factor for gastric cancer [36], and previous history of colorectal cancer, diabetes mellitus and gastric ulcer increased the risk of gastric cancer distinctly [37–39]. But they were excluded when this study analyzed the correlation between them and the risk of EGC, probably because their proportion was too small to correlate with the risk of EGC.

### Advantages and limitations

The greatest advantage of this study is that it screened the risk of EGC accurately and noninvasively. Some scholars have continuously studied medical instruments and detection reagents to improve the screening of EGC, and they applied the research results to the clinical gastroscopy and biopsy [24, 40]. A few researchers have combined genetics, proteomics and molecular biology to diagnose EGC [41, 42]. However, due to the restrictions of invasion, complexity, high cost or low compliance, these achievements have not been widely used in the clinical practice of screening for EGC. This study applied data mining methods to screen the risk of EGC in the light of noninvasive factors. Data mining methods obtained better predictions than traditional epidemic and health statistical methods when dealing with numerous factors and complicated relations among factors [22, 23]. Patients was initially screened by the optimal data mining models established, and then the high-risk patients screened were confirmed by further endoscopy plus pathology biopsy. This hierarchical screening strategy of EGC has high compliance and low cost, which will easily increase the screening coverage of EGC in clinical practice.

The limitations of this study include the patients from 26 hospitals, which participated in the project of the First Hospital Affiliated Guangdong Pharmaceutical University, slanted toward the narrow socioeconomic scale, limiting how these results could be generalized to more affluent populations. Furthermore, this study employed SMOTE to balance the training set to heighten the predictive performance of the models, but the data generated by SMOTE were not real data after all. Future researches will gather sufficient real data, the minority classe in particular, to further qualify the overall result. Ultimately, the effective prediction models performed will be applied to construct a cloud platform of screening for EGC to promote the clinical detection of EGC in future.

## Conclusions

This study utilized the data of noninvasive questionnaires and serological examinations, but the unpopulare and low compliable endoscopy plus pathology biopsy, to implement four models of screening for the risk of EGC. The three data mining models having better performances can be applied to assist clinicians hierarchical screening for the risk of EGC, which will improve the screening of EGC on a large scale. The data mining models may quickly assess the progression of gastric cancer, which will arise the attention of doctors and patients, then some proper measures would be taken to enhance the survival and life quality of the patients, especially when patients are predicted to be at high risk of EGC. This study found 16 crucial influence factors for the risk of EGC, such as occupations, HP infection, HP antibody, drinking hot water, eating pickled foods and so on. They are reminders to early prevention, early detection and early treatment of gastric cancer. This study may help clinical researchers in selecting and conducting the optimal predictive models, and assess important influence factors, to a great extent.

## Abbreviations

AUC: Area under the receiver operator characteristic curve
BMI: Body mass index
C5.0 DT: C5.0 decision tree
EGC: Early gastric cancer
HP: Helicobacter pylori
LR: Logistic regression
MLP: Multilayer perceptron
SMOTE: Synthetic minority oversampling technique
TAN: Tree augmented naive Bayesian network
